# Overexpression of *dilp2* causes nutrient-dependent semi-lethality in *Drosophila*

**DOI:** 10.3389/fphys.2014.00147

**Published:** 2014-04-16

**Authors:** Yukiko Sato-Miyata, Keigo Muramatsu, Masabumi Funakoshi, Manabu Tsuda, Toshiro Aigaki

**Affiliations:** ^1^Cellular Genetics Laboratory, Department of Biological Sciences, Tokyo Metropolitan UniversityTokyo, Japan; ^2^Department of Liberal Arts and Human Development, Faculty of Health and Social Services, Kanagawa University of Human ServicesYokosuka, Japan

**Keywords:** *Drosophila* insulin-like peptides, insulin-like growth factor signaling, hyperinsulinemia, growth regulation, autophagy, protein-to-carbohydrate ratio

## Abstract

Insulin/insulin-like growth factor (IGF) plays an important role as a systemic regulator of metabolism in multicellular organisms. Hyperinsulinemia, a high level of blood insulin, is often associated with impaired physiological conditions such as hypoglycemia, insulin resistance, and diabetes. However, due to the complex pathophysiology of hyperinsulinemia, the causative role of excess insulin/IGF signaling has remained elusive. To investigate the biological effects of a high level of insulin in metabolic homeostasis and physiology, we generated flies overexpressing *Drosophila insulin-like peptide 2* (*Dilp2*), which has the highest potential of promoting tissue growth among the *Ilp* genes in *Drosophila*. In this model, a *UAS-Dilp2* transgene was overexpressed under control of *sd-Gal4* that drives expression predominantly in developing imaginal wing discs. Overexpression of *Dilp2* caused semi-lethality, which was partially suppressed by mutations in the insulin receptor (*InR*) or *Akt1*, suggesting that *dilp*2-induced semi-lethality is mediated by the PI3K/Akt1 signaling. We found that *dilp2*-overexpressing flies exhibited intensive autophagy in fat body cells. Interestingly, the *dilp2*-induced autophagy as well as the semi-lethality was partially rescued by increasing the protein content relative to glucose in the media. Our results suggest that excess insulin/IGF signaling impairs the physiology of animals, which can be ameliorated by controlling the nutritional balance between proteins and carbohydrates, at least in flies.

## Introduction

In mammals, the peptide hormone insulin promotes glucose uptake in muscles and adipose tissues, induces cell growth and proliferation, and stimulates glyconeogenesis, lipogenesis, and protein synthesis (Saltiel and Kahn, 2001). The insulin/insulin-like growth factor (IGF) signal is evolutionally conserved throughout multicelluar organisms (Skorokhod et al., 1999). In insects, *Drosophila* has been extensively used as a model system to study insulin signaling, which plays an important role in regulating organ growth and the final size of the organism.

*Drosophila* possesses eight insulin-like peptides (*Dilps*), which can activate the *Drosophila* insulin receptor, InR (Brogiolo et al., 2001). Among the *Drosophila* insulin-like peptides (*Ilp*s), *dilp2* is the most highly expressed and it has the highest potential for promoting tissue growth (Ikeya et al., 2002; Rulifson et al., 2002; Broughton et al., 2005). It has been demonstrated that reduction of *dilp2* increases the content of the insect blood sugar, trehalose, in adult flies, suggesting that *dilp2* regulates glucose homeostasis in *Drosophila* as it also does in mammals (Broughton et al., 2008). Furthermore, reduction of *dilp2* expression has been shown to increase lifespan, indicating that *dilp2* plays an important role in lifespan determination (Broughton et al., 2008).

On the other hand, excess activation of insulin signaling could impair the physiology of organisms. In humans, it has been proposed that increased levels of insulin in the blood is a primary cause of Type 2 diabetes associated with hypertension and cancers (Novosyadlyy and LeRoith, 2010). In fact, hyperinsulinemia, which is an excessive level of insulin in the blood, is often seen in several metabolic diseases, such as Type 2 diabetes mellitus (Samuel and Shulman, 2012). However, the coexistence of hyperglycemia, insulin resistance, and other hormonal and metabolic changes in patients with Type 2 diabetes makes it difficult to understand the causative role of excess insulin signaling in the pathophysiology of hyperinsulinemia (Corkey, 2012). Several animal models for hyperinsulinemia have been developed by overexpressing InR or IGFR in some tissues, by the short-time administration of insulin, or by feeding animals a high-sugar diet (Musselman et al., 2011). Although these models have contributed to elucidating the molecular mechanisms that regulate insulin/IGF signaling, how hyperinsulinemia affects animal physiology has remained elusive.

It has been demonstrated that dietary composition also affects physiology and lifespan of individuals. In *Drosophila*, the balance of protein to carbohydrate intake is one of the critical determinants for lifespan and fecundity (Lee et al., 2008; Skorupa et al., 2008; Lushchak et al., 2012). For example, flies maintained with glucose-rich/protein-poor food generally become obese with age and exhibited a shorten lifespan and vise versa. Although insulin/IGF signaling plays crucial roles in regulation of glucose uptake, how the signal influences the dietary composition-dependent physiological changes is unclear.

To investigate how excess insulin affects insect physiology, we generated transgenic flies with a high level of *dilp2* and analyzed their phenotypes. Overexpression of *dilp2* increased the body size and caused semi-lethality. These phenotypes were partially suppressed by mutations in the insulin/IGF signaling pathway components, thereby suggesting that hyperactivation of the insulin/IGF signaling is toxic to flies. We found that *dilp2*-overexpressing flies exhibited intensive autophagy in fat body cells. Interestingly, increasing the protein content relative to glucose in the media partially rescued the *dilp2*-induced semi-lethality and autophagy. Our results suggest that excess insulin/IGF signaling impairs the physiology of animals, but it can be ameliorated by controlling the nutritional balance between proteins and carbohydrates, at least in flies.

## Materials and methods

### Fly stocks and media

*UAS-dilp2* (Brogiolo et al., 2001), *Akt1*^*1*^ (Stocker et al., 2002), and *InR*^*304*^ (Brogiolo et al., 2001) were kindly provided by Dr. E. Hafen. *PTEN*^*dj189*^ was a gift from Dr. D. Pan (Gao et al., 2000). *Tor*^*K17004*^(Oldham et al., 2000), *S6K*^*07064*^ (Montagne et al., 1999), and *M{3xP3-RFP.attP}ZH-51D* and *M{3xP3-RFP.attP}ZH-68E* (Bischof et al., 2007) were obtained from the Bloomington Stock Center. Flies were reared at 25°C on a standard cornmeal medium [3.6% neutralized yeast (Asahi Breweries, LTD. Y-4), 8.1% cornmeal, 10% glucose, and 0.7% agar] with propionic acid and *n*-butyl *p*-hydroxybenzoate as mold inhibitors, unless otherwise stated. We used different medium for the convenience of preparation. *Drosophila* Instant Medium (Formula 4-24, Carolina Biological. Supply, Burlington, NC) was used to as a basal medium to prepare media containing different concentration of yeast extracts: 2 g of *Drosophila* Instant Medium was mixed with 5 ml of Bacto™ Yeast Extract (Difco Laboratories, Detroit, MI, USA) dissolved in water at four different concentrations (0, 10, 20, and 40 g/L). Standard cornmeal agar medium was used to prepare media containing glucose at four different concentrations (0, 100, 200, and 300 g/L).

### Genetic interaction experiments

To facilitate genetic interaction experiments, we generated a stock, *sd-Gal4/sd-Gal4; UAS-dilp2/TM6B, tub-Gal80*, in which GAL4-dependent expression of *dilp2* is repressed by GAL80. The stock is convenient to test the effects of mutations on the phenotype caused by overexpression of *dilp2*. To make an internal control, we made flies heterozygous for a mutation with a homologous chromosome marked with RFP: second chromosome-linked mutations (*Tor*^*K17004*^ and *PTEN*^*dj189*^) and third chromosome-linked mutations (*Akt1*^*1*^, *InR*^*304*^, and *S6K*^*07064*^) were crossed to *M{3xP3-RFP.attP}ZH-51D* and *M{3xP3-RFP.attP}ZH-68E*, respectively. The F1 progenies (mutations/*3xP3-RFP*) were crossed to *sd-Gal4/sd-Gal4; UAS-dilp2/ TM6B, tub-Gal80* flies. Numbers of resulting progenies (*sd-Gal4*/+; *UAS-dilp2*/+ with mutations) and their sibling controls (*sd-Gal4*/+; *UAS-dilp2*/+ with *3xP3-RFP*) were counted and calculated relative viabilities.

### Measurement of body weight and wing size

The adult flies were weighed using an Analytical Semi-Micro Balance (A&D Company, Tokyo, Japan). To measure wing size, the right wings of the adult flies were torn off by using forceps and mounted onto a microscopic slide using a drop of Fly Line Dressing (TIEMCO, Tokyo, Japan), a silicone grease with very low surface tension (Tsuda et al., 2010). The wings were photographed using a MZ APO stereomicroscope (Leica, Wetzlar, Germany) equipped with a DP50 digital camera (Olympus, Tokyo, Japan) at a constant magnification. The areas of the wings were measured by using ImageJ software (NIH).

### Quantitative real-time PCR

Total RNA from the adult flies was extracted using TRIzol^®^ (Qiagen, Valencia, CA, USA) and it was reverse-transcribed using ReverTra Ace^®^ (Toyobo, Osaka, Japan). Quantitative-PCR reactions were carried out using SYBR^®^ Premix Ex Taq™ (Takara Bio, Otsu, Japan).

### Western blot analysis

The adult flies were homogenized in SDS sample buffer (12.5 mM Tris (pH 6.8), 20% glycerol, 4% SDS, 2% 2-mercaptoethanol, and 0.001% bromophenol blue) and boiled for 10 min at 95°C. The samples were separated by 10% SDS-PAGE and transferred to PVDF membranes (GE Healthcare, Buckinghamshire, UK). After blocking with 5% bovine serum albumin (Sigma-Aldrich, St. Louis, MO, USA), the membranes were incubated with a primary antibody in Tris-buffered saline (TBS) containing Tween-20 (TBST) overnight at 4°C and then with a secondary antibody in TBST for 1 h at 25°C. The signals were detected with an ECL-plus kit (GE Healthcare). As primary antibodies, rabbit anti-phospho-Akt antibody (Cell Signaling Technology, Danvers, MA, USA), rabbit anti-phospho-p70 S6 kinase (Cell Signaling Technology), and mouse anti-α-tubulin (Sigma-Aldrich) were used at dilutions of 1:1000, 1:1000, and 1:5000, respectively. HRP-conjugated anti-rabbit IgG (Cell Signaling Technology) and HRP-conjugated anti-mouse IgG (GE Healthcare) were used as secondary antibodies at dilutions of 1:2000 and 1:1000, respectively.

### Liquid chromatography coupled to tandem mass spectrometry (LC-MS/MS)

LC-MS/MS was used to determine the concentrations of glucose, trehalose, glucose metabolites, and free amino acids. Ten female flies were weighted and homogenized with 75% acetonitrile on ice. The homogenates were centrifuged for 10 min at 2400 × g and the supernatant was re-centrifuged for 10 min at 1200 × g. The supernatant was evaporated and dissolved to mobile phase (10 mM DBAA, Tokyo Chemical Industry, Tokyo, Japan), in H_2_O (pH 4.75). After centrifuging at 1200 × g to remove residue, the supernatants were collected and used for metabolome analysis with a Waters LC-QTofMS system composed of LC (Acquity UPLC) and MS (Xevo™ QTofMS) (Waters, Milford, MA, USA). The metabolites were separated on an Acquity^®^ UPLC HSS T3 column (2.1 Å ~ 100 mm, 1.8 μm; Waters). For the carbon metabolites, the columns were equilibrated with 10 mM DBAA (Tokyo Chemical Industry) in H_2_O (pH 4.75), and the compounds were eluted with an increasing gradient of acetonitrile. The total run time was 20 min. The MS system was equipped with a dual electrospray ionization probe and operated in the negative ion mode with the source temperature at 120°C. For the amino acids, the analytes were separated by a gradient of mobile phase ranging from water containing 0.05% acetic acid to methanol over a 15 min run. The capillary voltage and the cone voltage for electrospray ionization was maintained at 0.7 kV and 15 V for negative mode detection and at 0.7 kV and 13 V for positive mode detection, respectively. The source temperature and the desolvation temperature were set at 120 and 350°C, respectively. Nitrogen was used as both the cone gas (50 l/h) and the desolvation gas (600 l/h) and argon was used as the collision gas. For accurate mass measurement, the mass spectrometer was calibrated with sodium formate solution (range m/z 50–1000) and monitored by the intermittent injection of the lock mass leucine enkephalin ([M + H]+ = 556.2771 m/z and [M − H]− = 554.2615 m/z) in real time. The MS data were analyzed using QuanLynx™ (Waters). The compounds were identified based on their retention time, their m/z ratio, and the MS/MS spectrum of standard reference materials.

### Measurement of protein content

The concentration of soluble protein was measured using the Bio-Rad protein assay reagent. At least three trials were carried out for each genotype.

### Triglyceride measurement

Ten adult flies were weighed and homogenized in 1% Triton-X. The homogenates were heated for 10 min at 70°C and stored at −80°C. After thawing on ice, the samples were centrifuged for 10 min at 14,700 × g at 25°C. The amount of triglycerides (TAG) was determined using a Serum Triglyceride Determination Kit (TR0100; Sigma–Aldrich, St. Louis, MO, USA). All data were normalized with soluble protein contents in homogenates. At least three samples were used to determine the average amount of triglycerides.

### Autophagy staining

The fat bodies were dissected from the early third larvae in PBS and incubated with LysoTracker^®^ Red DND-99 (Molecular Probes^®^, Life Technologies, Grand Island, NY, USA) at a 1:1000 dilution in PBS for 1 min at room temperature. After a brief wash in PBS, the sample was observed under a Nikon C1 laser scanning confocal microscope.

## Results and discussion

### Overexpression of *dilp2* reduced the egg-to-adult viability of the flies

**Figure 1:**
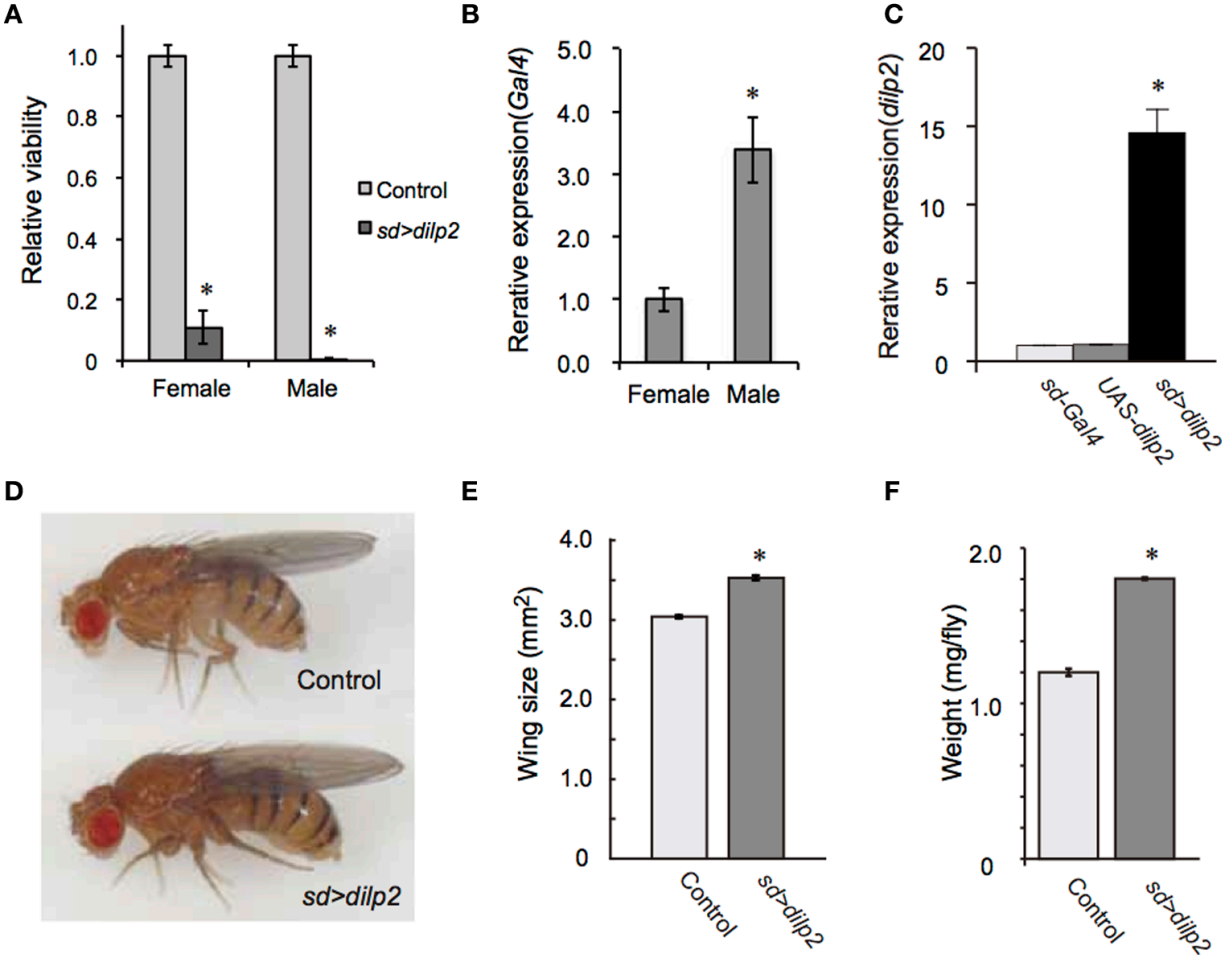
**Overexpression of *dilp2* increases body size and causes semi-lethality**. Relative viability of *dilp2*-overexpressing flies **(A)**. Flies homozygous for *sd-Gal4* were crossed with *UAS-dilp2*/*3xP3-RFP* males, and the number of flies expressing RFP served as an internal control; those not expressing RFP are expressing *dilp2*. Expression level of Gal4 in *sd-Gal4* male is much higher than that of *sd-Gal4* female **(B)**. Overexpression of *dilp2* caused semi-lethality for both males and females. The relative expression level of *dilp2* mRNA in the *dilp2*-overexpressing flies (*sd* > *dilp2*) and the parental lines (*sd-Gal4* and *UAS-dilp2*) served as controls and was determined by real-time RT-PCR **(C)**. *dilp2*-overexpressing flies have increased body size **(D)**, increased wing area by 17% **(E)**, and increased weight by 50% **(F)**. Student *t*-test was performed to analyze statistical significance. ^*^p < 0.01.

To generate a transgenic fly model of hyperinsulinemia, we overexpressed *dilp2* which is known to promote tissue growth in *Drosophila*. We found that flies show high lethality when *dilp2* was overexpressed ubiquitously using *actin5c-Gal4*, suggesting that high levels of Dilp2 are toxic to flies. However, the reason for this lethality was not understood. An alternative driver, which gives a milder phenotype, would be useful to investigate the developmental toxicity associated with *dilp2* overexpression. We tested several *Gal4* lines, and found that *sd-Gal4*, which predominantly expresses *Gal4* in developing imaginal wing discs, was an appropriate driver. When *sd-Gal4* was crossed with *UAS-dilp2*, the number of progenies from the cross was significantly less than the number of progenies obtained from the parental lines, thereby suggesting that overexpression of *dilp2* was toxic to flies. To more quantitatively determine the viability, the number of progenies were compared between the *dilp2*-overexpressing flies and their siblings bearing the *M{3xP3-RFP.attP}ZH-68E* chromosome expressing red fluorescence protein (RFP) under the control of an artificial *3xP3* promoter (Bischof et al., 2007). Female flies homozygous for *sd-Gal4* were crossed to *UAS-dilp2M{{3xP3-RFP.attP}ZH-68E* heterozygous males and the number of adult flies was counted. In theory, one half of the progeny inherit and express *UAS-dilp2* under control of *sd-Gal4*, and the other half, serving as an internal control, carry an RFP-bearing chromosome. The number of *dilp2*-overexpressing flies was markedly reduced compared to that of the control flies (Figure 1A). Both sexes are semi-lethal, but the effects were more severe in the males than in the females. This is likely due to the dosage compensation mechanism, since *sd-Gal4* is an X-linked transgene, which drives expression of *dilp2* two-times higher in males (Figure 1B).

Using female flies that survived to adult stage, we determined the expression level of *dilp2* in females. Quantitative real-time PCR revealed that the levels of *dilp2* mRNA in the adult female increased 16-times higher than that of the control, suggesting that overexpression of *dilp2* occurred and is toxic to fly development (Figure 1C).

We also noticed that the body size of the *sd* > *dilp2* flies that survived to the adult stage was larger than that of the control (Figure 1D). To compare the body size more precisely, wing size was measured as described in the Materials and Methods section. The wing size of the *sd* > *dilp2* flies was increased by 17% compared to the wing size of the control flies (Figure 1E). In addition, the body weight of the *dilp2*-overexpressing flies increased by 50% compared to the control flies (Figure 1F). These results indicated that overexpression of *dilp2* caused semi-lethality, but promoted growth for the survivors.

### The PI3K/AKt1pathway mediates *dilp2*-induced semi-lethality

**Figure 2:**
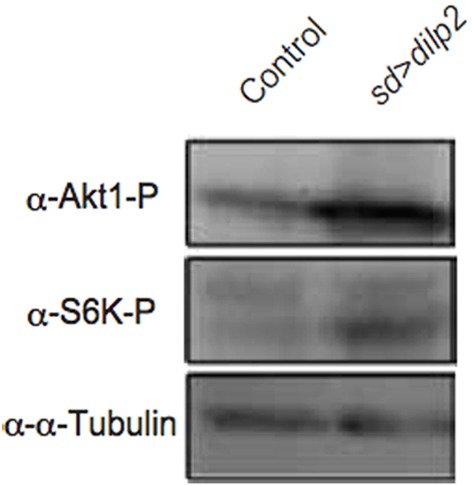
***dilp2*-overexpression activates Akt1 and S6 kinase (S6K)**. The amount of phosphorylated Akt1 and S6K were analyzed by western blots. α-tubulin was used as a loading control.

**Figure 3:**
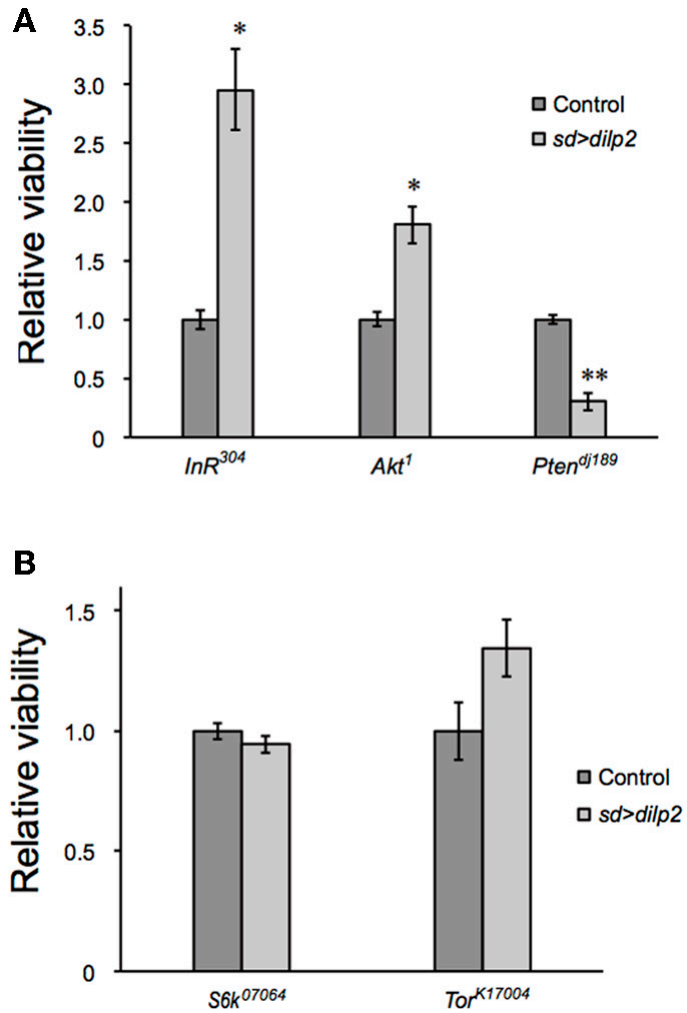
**Genetic manipulation of InR/Akt1/PI3K modifies the *dilp2*-induced lethality**. Relative viability of the *sd* > *dilp2* flies was determined in combination with loss of function mutations in *InR*, *Akt1*, or *PI3K*, which are components of the insulin/IGF signaling pathway. *dilp2*- induced semi-lethality was markedly improved by reduction of *InR* or *Akt1*, but enhanced by reduction of *PTEN* **(A)**. Relative viability of the *sd* > *dilp2* flies in combination with loss-of-function mutations in *Tor* and *S6K* **(B)**. The Tor/S6K pathway may not contribute to mediating the semi- lethality of *dilp2*-overexpressing flies. Student *t*-test was performed to analyze statistical significance. ^*^p < 0.05, ^**^p < 0.01.

*dilp2* stimulates the PI3K/Akt1 pathway through insulin receptor (InR) activation. Western blotting analysis revealed that the active form of Akt1 was significantly increased in the *dilp2*-overexpressing flies compared to the control, indicating that overexpression of *dilp2* indeed activates the PI3K/Akt1 signaling (Figure 2 upper panel). To further confirm this, we investigated whether the mutations in the pathway components had an effect on *dilp2*-induced semi-lethality. We found that, for both males and females, the heterozygous flies that carry a loss-of-function mutation in InR or Akt1 showed higher levels of viability compared to the control flies (Figure 3A). On the other hand, a loss-of-function mutation in Ptne, a negative regulator of PI3K, further reduced the viability of *dilp2*-overexpressing flies (Figure 3A). These results suggest that *dilp2*-induced semi-lethality is mediated by the PI3K/Akt1 signaling. Overexpression of *IlpP2* also activated the Tor/S6K signaling, a downstream signal component of the PI3K/Akt1 signaling, since the level of phosphorylated S6K was increased (Figure 2 middle panel). However, interestingly, the loss-of-function mutations in Tor or S6K had no effect on the reduced viability of the *dilp2*-overexpressing flies, suggesting that the Tor/S6K signaling is irrelevant to the semi-lethality or the single copies of mutations were not sufficient to suppress the *dilp2*-induced semi-lethality (Figure 3B).

### Glucose and lipid metabolism in *dilp2* overexpressing flies

**Figure 4:**
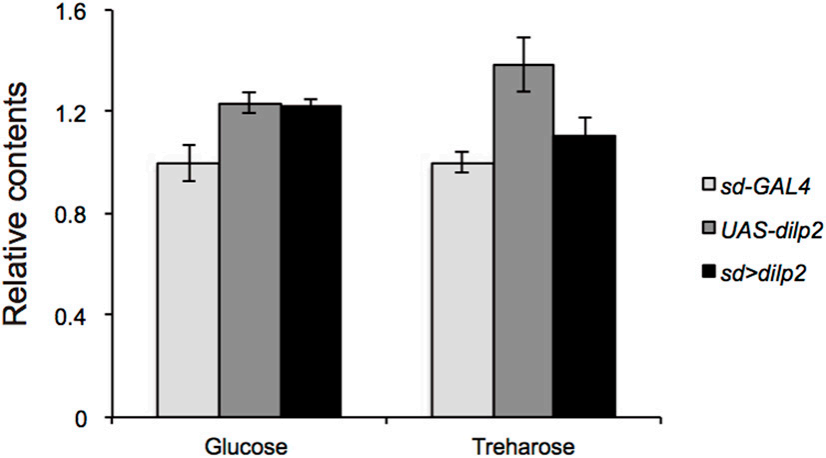
**Trehalose and glucose content in *dilp2*-overexpressing flies**. Relative amount of glucose and trehalose in the *sd* > *dilp2* flies was calculated based on the values of a parental line (*sd-Gal4*) and indicated as the mean ± SE of at least three samples with a minimum of five flies per group. There was no significant difference in the amount of these sugars.

**Figure 5:**
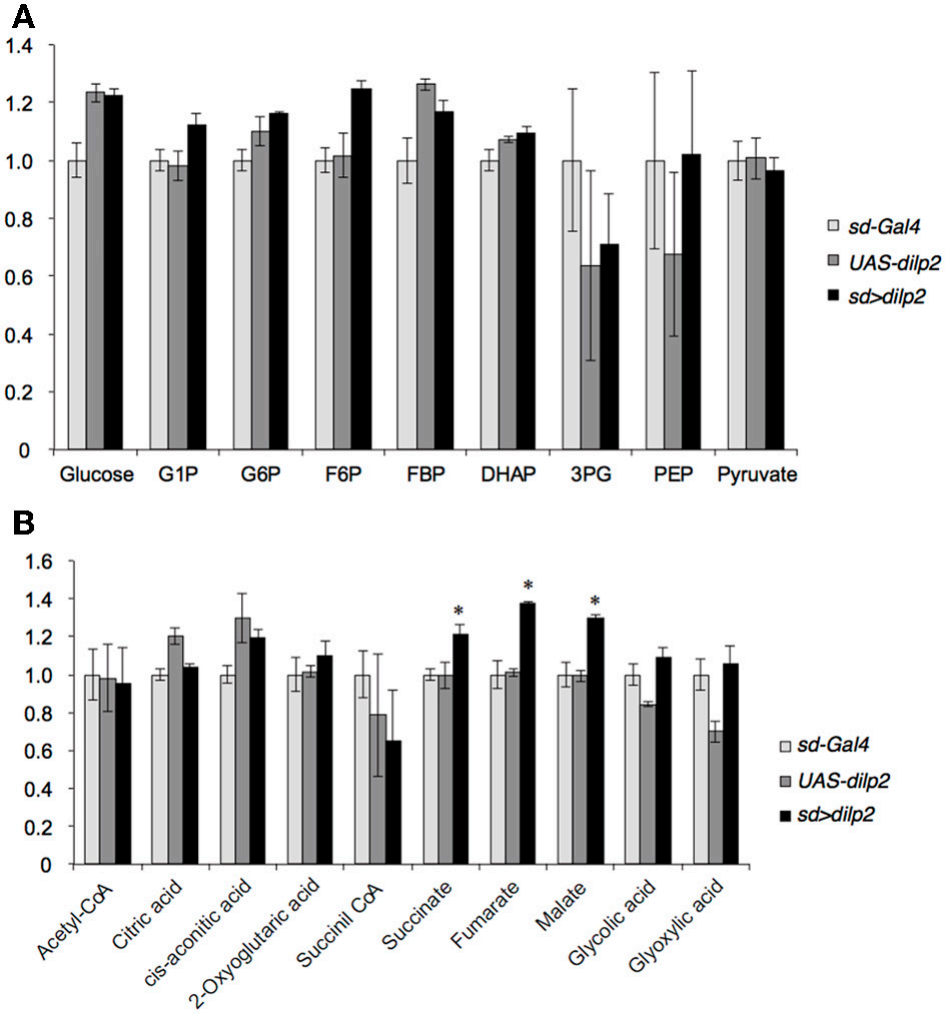
**Glucose metabolism in *dilp2*-overexpressing flies**. Glycolysis **(A)** and TCA cycle **(B)** metabolites were analyzed by using LC-MS/MS in negative mode. Relative amounts of metabolites in the *sd* > *dilp2* flies were calculated based on the values of the parental lines (*sd- Gal4*) and shown as the mean ±S.E of at least three samples with a minimum of five flies per group. G6P: glucose 6-phosphate;F6P: fructose 6-phosphate; F1,6BP: fructose 1,6-bisphosphate; 2/3-PG: 2/3-phophoglycerate; PEP: phosphoenolpyruvate. Student *t*-test was performed to analyze statistical significance. ^*^p < 0.05

Next, we examined whether overexpression of *dilp2* disrupts glucose homeostasis and metabolism. We first measured the concentration of glucose and trehalose, the major blood sugars in insects. There was no significant difference in the concentration of these sugars between the *dilp2*-overexpressing flies (*sd* > *dilp2*) and the parental lines (*sd-Gal4* or *UAS-dilp2*) as the controls (Figure 4). We also measured the amounts of glycolysis and TCA cycle metabolites using liquid chromatography coupled to tandem mass spectrometry (LC-MS/MS) with ion-pair reagents (Figures 5A,B). There was no significant difference in the amounts of glycolytic metabolites between the *dilp2*-overexpressing flies (*sd* > *dilp2*) and the parental lines (*sd-Gal4* or *UAS-dilp2*) as the controls. These results suggest that glucose homeostasis itself was unaffected by overexpression of *dilp2*. For the TCA cycle metabolites, the amount of succinate, fumarate, and malate was slightly increased in the *dilp2*-overexpressing flies. Since fumarate and malate have been shown to extend lifespan in *Caenorhabditis elegans* (Edwards et al., 2013), overexpression of *dilp2* might have affected the energy metabolism associated with lifespan determination.

**Figure 6:**
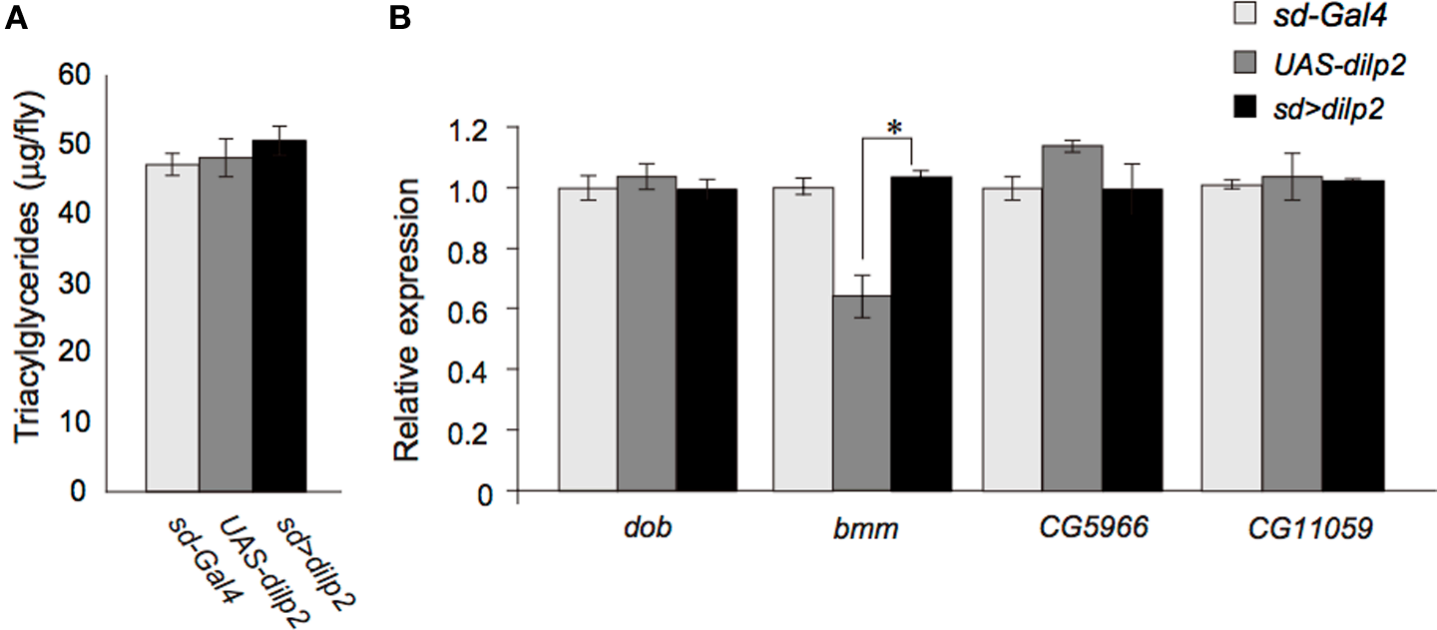
**Lipid metabolism in *dilp2*-overexpressing flies**. Comparison of TAG levels between the *dilp2*-overexpressing flies and the parental lines as controls **(A)**. Relative mRNA levels of four major genes involved in lipid catabolism in *Drosophila*, *doppelganger von brummer* (*dob*), *brummer* (*bmm*), *CG5966*, and *CG11055* **(B)**. Results are shown as the mean ± SE of at least three experiments. ANOVA with Tukeys HSD was performed to analyze statistical significance. ^*^p < 0.01.

Insulin/IGF signaling might down-regulate lipid catabolism via regulation of lipases (Xu et al., 2012). Thus, we next measured the lipid content in the *dilp2*-overexpressing flies and the control parental lines. There was no significant difference in the level of triacylglycerol, the most abundant of the storage lipids (Figure 6A). In addition, quantitative real-time PCR analysis revealed that the expression levels of four major *Drosophila* lipases, *doppelganger von brummer* (*dob*), *brummer* (*bmm*), *CG5966*, and *CG11055* (Gronke et al., 2005) were not altered in the *dilp2*-overexpressing flies (Figure 6B). Expression level of *bmm* in the parental line, *UAS-dilp2* was significantly lower than those of another parental line *sd-Gal4* and of their progeny *sd* > *dilp2*. There might be unknown mechanisms that downregulate *bmm* expression in the background of *UAS-dilp2* line. We also compared the expression levels of *bmm* between *sd* > *dilp2* and *sd-Gal4*, *3xP3-RFP* flies, and found that there was no significant change in *bmm* expression level when *dilp2* was overexpressed (data not shown).

These results suggested that overexpression of *dilp2* does not affect the lipid storage and catabolism in flies.

### Nutrient dependent effects of *dilp2*-overexpression

**Figure 7:**
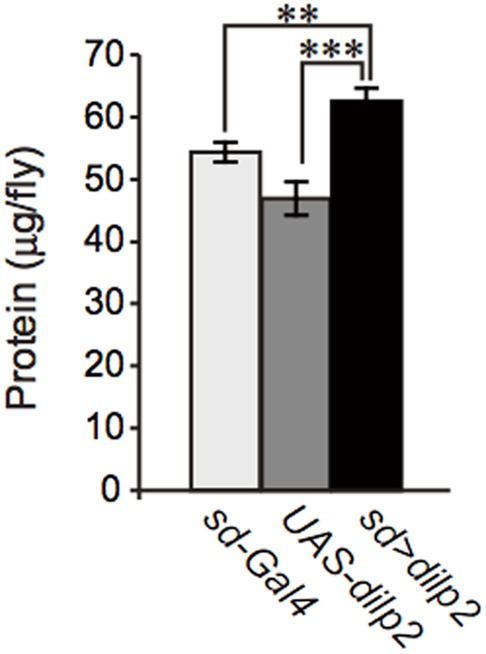
**Protein content in *dilp2*-overexpressing flies**. The protein content was significantly increased in the *dilp2*- overexpressing flies (*sd* > *dilp2*) compared to the parental lines (*sd-Gal4* and *UAS-dilp2*) as controls. Results are shown as the mean ± SE of at least three experiments. Student *t*-test was performed to analyze statistical significance. ^**^p < 0.01; ^***^p < 0.001.

**Figure 8:**
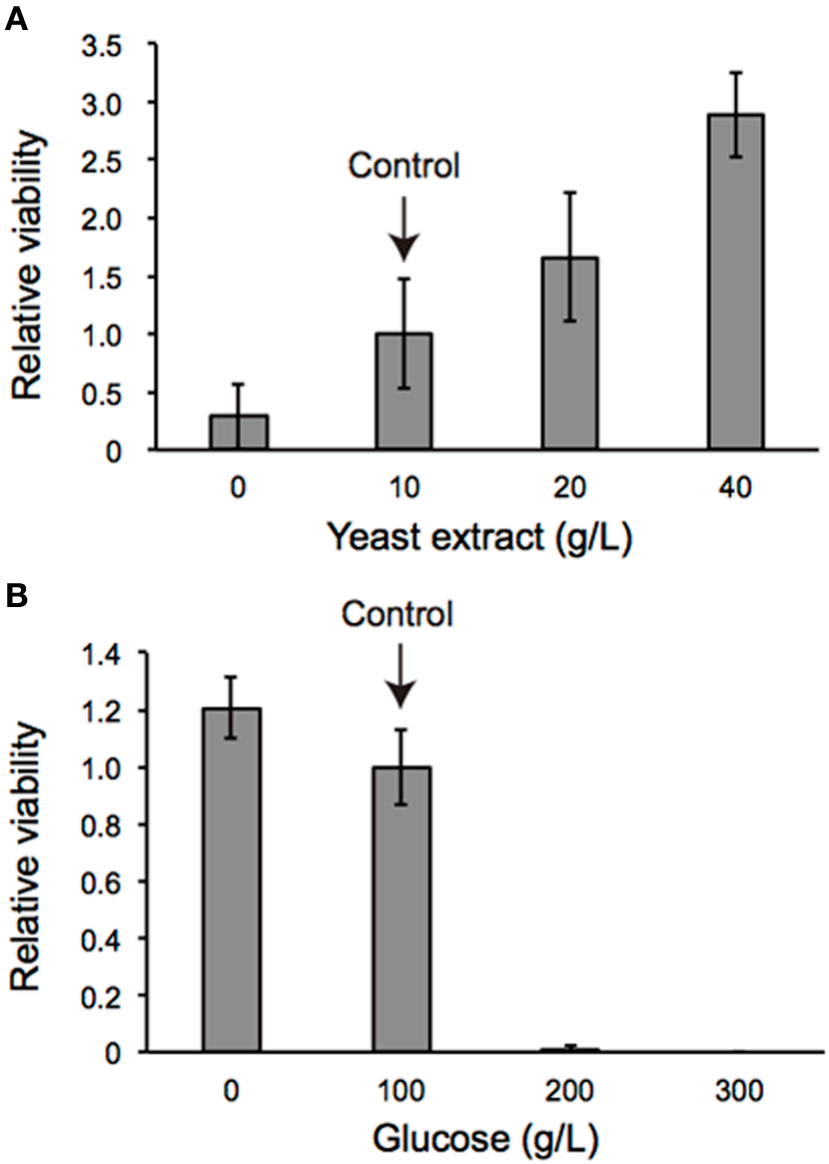
**Viability of *dilp2*-overexpressing flies depends on nutritional conditions**. *Dilp2*-overexpressing flies (*sd* > *dilp2*) were reared on media supplemented with various concentrations of yeast extract **(A)** or glucose **(B)**. Relative viability of the *dilp2*-overexpressing flies (*sd* > *dilp2*) for each medium was calculated based on the number of control siblings.

**Figure 9:**
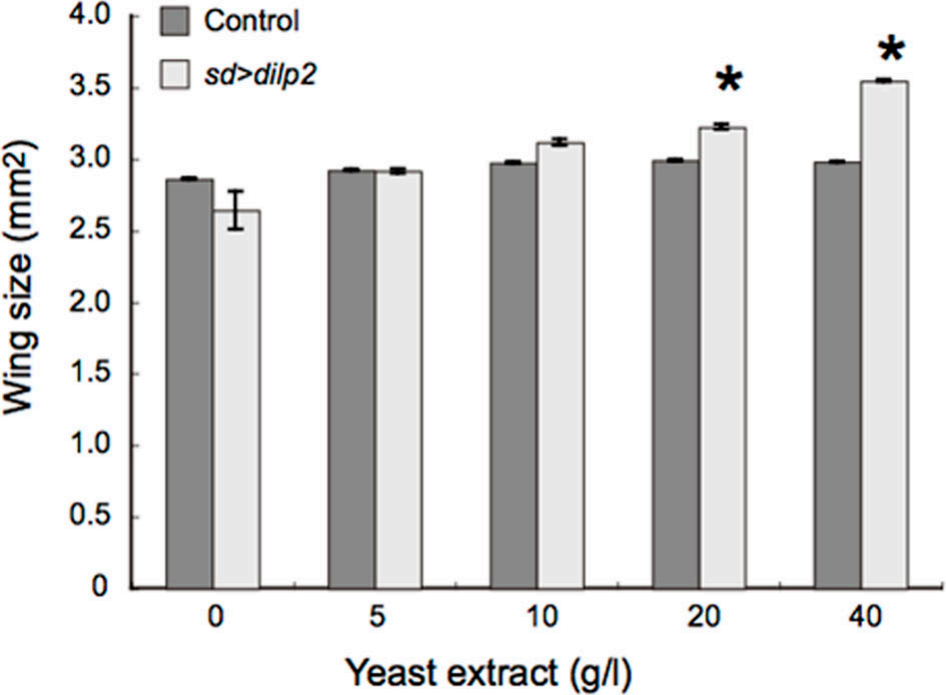
**Effects of protein-rich media on the wing size of *dilp2*-overexpressing flies**. *dilp2*-overexpressing flies (*sd* > *dilp2*) were reared on media supplemented with various concentrations of yeast extract and their wing sizes were compared with those of the internal control flies. Mean wing area was positively correlated with the concentration of yeast extract in the media. Student *t*-test was performed to analyze statistical significance. ^*^p < 0.05.

**Figure 10:**
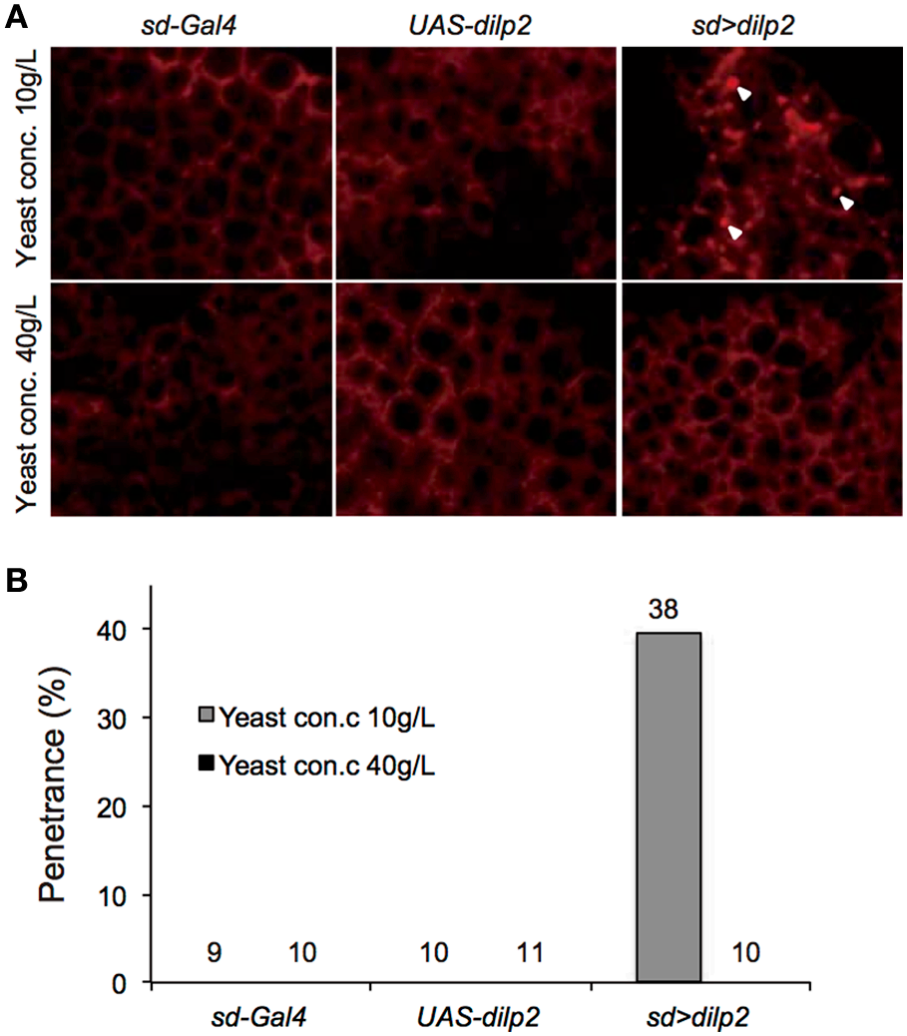
**Intensive autophagy in *dilp2*-overexpressing flies**. Fat bodies were dissected from early third instar larvae overexpressing *dilp2* (*sd* > *dilp2*) and stained with LysoTracker Red DND-99 **(A)**. When the flies were reared on standard media containing 10 g yeast/L, strong punctate signals (arrowheads), corresponding to autophagy were observed in the *sd* > *dilp2* flies (upper right panel) but not in the parental lines (*sd-Gal4* or *UAS-dilp2*) as the controls (upper left and middle panels). Autophagy was not detectable in the *sd* > *dilp2* flies when they were reared on protein-rich media containing 40 g yeast/L (lower panels). The penetrance (frequency) of ectopic autophagy in *sd* > *dilp2* flies **(B)**. Numbers of samples observed were indicated above each bar.

We also examined the protein content per fly and found that the *dilp2*-overexpressing flies contain more protein than the parental control lines (*sd-Gal4* and *UAS-dilp2*), thereby suggesting that protein synthesis is elevated in *sd* > *dilp2* flies (Figure 7). Interestingly, the viability of the *dilp2*-overexpressing flies increased depending on the concentration of the yeast extracts in the media (Figure 8A; Spearman's rank correlation coefficient = 0.79, *p* < 0.0003). The relative viability of the *dilp2*-overexpressing female flies was only 2% in the medium without yeast extract, whereas it was 16.6% in the medium containing 40 g/L yeast extract. In addition, increasing protein content in the media significantly increased the mean wing area of the *dilp2*-overexpressing flies, while it did not affect the wing size of the control flies (Figure 9). These results indicated that to support their development the *dilp2*-overexpressing flies require more protein as a nutrient than the control flies. It is possible that a shortage of protein sources occurs in the *dilp2*-overexpressing flies. To test this hypothesis, we examined whether autophagy occurs in the fat bodies dissected from the early third instar larvae. A large number of autophagy positive-cells were observed in the fat bodies of the *dilp2*-overexpressing flies While no autophagy positive-cell was found in the fat bodies of the control flies, (Figure 10). Increasing protein content in the media significantly suppressed the *dilp2*-mediated autophagy, suggesting that the *dilp2*-overexpressing flies were suffering from an insufficiency of protein sources. Namely, the shortage of protein sources could be one of the reasons for *dilp2*-induced semi-lethality.

**Figure 11:**
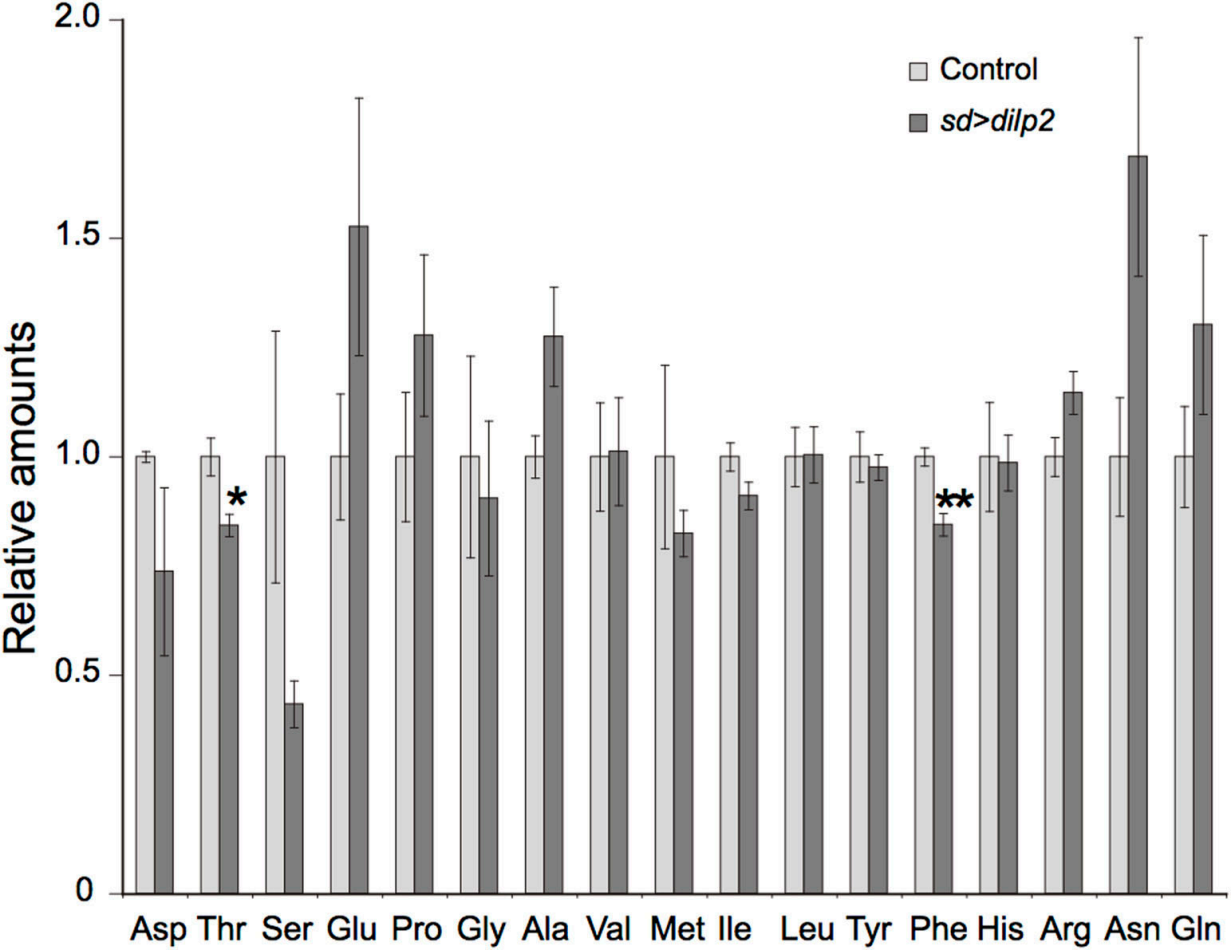
**Amino acid content in *dilp2*-overexpressing flies**. A free amino acids in the adult flies were quantified by using an LC-MS/MS in positive mode. Relative amounts of each amino acid in *sd* > *dilp2* flies were calculated based on those in the *sd* > *RFP* flies as a control, and are shown as the mean ± SE of at least three experiments with a minimum of five flies per group. Student *t*-test was performed to analyze statistical significance. ^*^p < 0.05, ^**^p < 0.01.

Overexpression of *dilp2* might cause chronic deficiency of amino acids. To examine this possibility, we quantified free amino acids in the *sd* > *dilp2* and the *sd* > *RFP* flies using an LC-MS/MS. Although the relative amount of threonine and phenylalanine were significantly different between the two groups, all changes were subtle and are unlikely to affect the viability (Figure 11). These results suggested that survivors of the *dilp2*-overexpressing flies were maintaining normal amino acid homeostasis probably though inducing ectopic autophagy. However, considering the semi-lethality of the animals, those failed to maintain amino acid homeostasis might have died earlier during development.

### A protein-to-carbohydrate ratio is critical for the survival of *dilp2*-overexpressing flies

**Figure 12:**
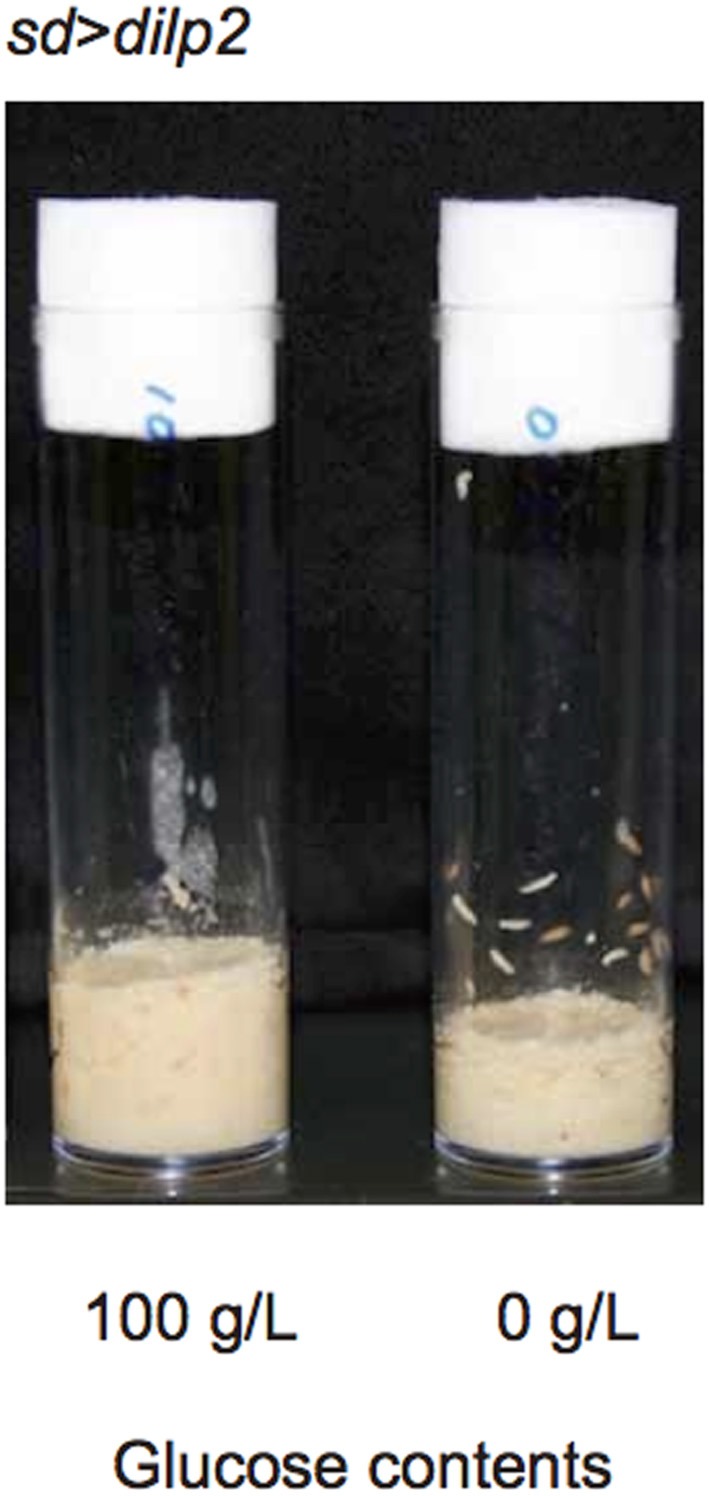
***dilp2*-overexpressing flies develop faster in media with reduced glucose**. Overexpression of *dilp2* retarded development of flies when they were reared on the standard media containing 100 g/L glucose (left). However, flies develop faster when kept in glucose-deprived media (right). The mean (±SE) durations of egg-to-early pupa was 106.3 ± 1.3 and 82.6 ± 1.3 h, with control media and glucose-deprived media, respectively (Student *t*-test: *p* < 0.001).

The occurrence of autophagy in *dilp2*-overexpressing flies suggested that the animals were suffered from the shortage of amino acids. It has been demonstrated that a protein-to-carbohydrate ratio can affect the lifespan and the fecundity of flies (Lee et al., 2008). It is possible that overexpression of *dilp2* affected the optimal ratio of these nutrients for the flies' development. Thus, we examined whether the glucose content of the fly food could modify the *dilp2*-induced semi-lethality. Increasing glucose concentration in the media significantly reduced the viability of the *dilp2*-overexpressing flies, indicating that overexpression of *dilp2* enhances the susceptibility to glucose. On the other hand, decreasing glucose content in the media significantly improved the viability of the *dilp2*-overexpressing flies (Figure 8B Spearman's rank correlation coefficient = −0.86, *p* < 0.0001). Interestingly, the *dilp2*-overexpressing flies kept in a glucose-reduced condition grew significantly faster than those reared on standard media (Figure 12). The mean (±SE) durations of egg-to-early pupa was 106.3 ± 1.3 and 82.6 ± 1.3 h, with control media and glucose-deprived media, respectively (Student *t*-test: *p* < 0.001). These results strongly suggested that a high-protein low-carbohydrate diet is optimal for the viability of *dilp2*-overexpressing flies.

In this study, we demonstrated that overexpression of *dilp2* severely decreases the egg-to-adult viability of flies and induced a high frequency of ectopic autophagy in fat bodies of early third instar larvae. As in mammalian cells, nutrient starvation induces autophagy through inhibition of Tor activity in *Drosophila* (Scott et al., 2004). However, the *dilp2*-overexpression-dependent autophagy does not seem to be regulated by the down-regulation of Tor, since S6K, a downstream target of Tor, was strongly activated (Figure 2). Activated S6K may execute autophagy, since expression of activated S6K increases starvation-induced autophagy in the absence of Tor in *Drosophila* (Scott et al., 2004). It is possible that a hyperactivation of S6K in the *dilp2*-overexpressing flies might contribute to promoting autophagy. In isolated rat hepatocytes, some amino acids inhibit induction of autophagy in an mTor (mammalian Tor) independent manner (Kanazawa et al., 2004). Therefore, a shortage of protein sources may induce autophagy directly. The *dilp2*-overexpression-dependent autophagy was reverted by a high-protein diet, suggesting that the nutritional condition is critical for survival of *dilp2*-overexpressing flies. It has been demonstrated that flies cultured on nutrient-rich food contain a significantly high level of secreted *dilp2* compared to flies cultured on nutrient-deprived food, indicating that flies can sense the nutrient availability and modulate their insulin secretion accordingly (Geminard et al., 2009). Our results suggest that excess insulin/IGF signaling impairs the physiology of animals, which can be ameliorated by controlling the nutritional balance between proteins and carbohydrates, at least in flies.

### Conflict of interest statement

The authors declare that the research was conducted in the absence of any commercial or financial relationships that could be construed as a potential conflict of interest.
